# Correction: Mapping the probability of forest snow disturbances in Finland

**DOI:** 10.1371/journal.pone.0257749

**Published:** 2021-09-17

**Authors:** Susanne Suvanto, Aleksi Lehtonen, Seppo Nevalainen, Ilari Lehtonen, Heli Viiri, Mikael Strandström, Mikko Peltoniemi

There are panels missing from Fig 5. Please see the correct Fig here.

**Fig 5:**
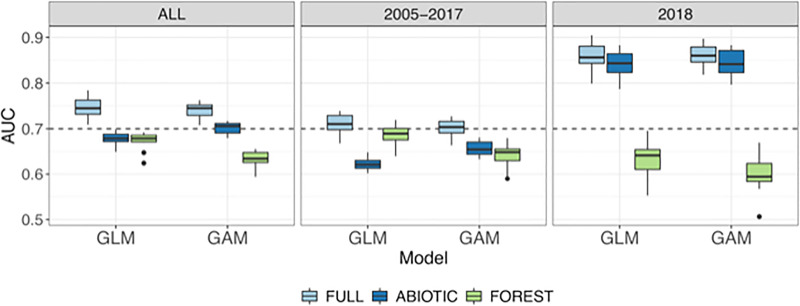
Cross-validation results for GLM and GAM with different predictor sets and for different time periods. Dash line shows the AUC = 0.7 threshold for acceptable level of discrimination between cases and non-cases.
