# Supplementary material for: Mapping the probability of forest snow disturbances in Finland
Source: PLoS One. 2021 Jul 29;16(7):e0254876. doi: 10.1371/journal.pone.0254876 (PMC8321231; doi:10.1371/journal.pone.0254876)
Supplement: S1 File — (PDF) [file pone.0254876.s001.pdf]

Supplementary material: Details of model selection

Suvanto et al. Mapping the probability of forest snow disturbances in Finland

1. Summary

The model predictors were chosen based on:

1. existing understanding of snow damage dynamics,
2. availability of national extent GIS-data to be used for map prediction,
3. statistical significance of highest order terms in model, requiring significance on the level of $p < 0.01$.
4. improvement in AIC when comparing alternative models and
5. collinearity between predictors, generalized variation inflation factor (GVIF) < 4.

Table 1: Variables considered in model selection and reasons for exclusion from the final model (see numbering above).

variable_name	description	included	reason
species_spruce	Dominant species spruce (ref.class=pine)	yes	–
species_other	Dominant species other (ref.class=pine)	yes	–
dbh	Diameter at breast height (DBH)	yes	–
basalarea	Basal area	yes	–
north	North boreal zone (0/1)	yes	–
snowload_longterm	Longterm average of max. snow load	yes	–
snowload	Snowload	yes	–
relelev_1km	Relative elevation	yes	–
dem	Altitude	yes	–
species_other:dbh	Interaction: Species, other x DBH	yes	–
species_spruce:north	Interaction: Species, spruce x North boreal zone	yes	–
species	Dominant species (ref.class=pine, 2=spruce, 3=other)	no	3
harvennus_yli5v	Thinning, > 5 yr	no	3, 4
ensiharvennus_yli5v	Precommercial thinning, >5 yr	no	3, 4
taimihoito_yli5v	Tending of seedling stands, >5 yr	no	2
keskipit	Average tree height	no	5, 3, 4
kptyyp_2class	Site type	no	3, 4
species:basalarea	Interaction: Species x Basal area	no	3
species:dbh	Interaction: Species x DBH	no	3
snowload:north	Interaction: Snowload x North boreal zone	no	3, 4
north:dbh	Interaction: North x DBH	no	3
north:species	Interaction: North x Species	no	3
snowload_longterm:species	Interaction: Longterm snowload x Species	no	3, 4
n_species	Number of species	no	2, 3
max_sp_percent	Share of BA by dominant species	no	3
shannon_div	Shannon diversity index, calculated from species BA	no	2

For continuous variables with non-negative values log transformation was tested and kept in the model if produced a lower AIC.

Results for the variables not included in the final model are presented below.

2. Thinning, > 5 years

Variable describes whether some type of thinning has been carried out at the stand more than five years ago (TRUE/FALSE).

Table 2: Model with variable "Thinning, > 5 years" (harvennus_yli5v)

	Estimate	Std. Error	z value	Pr(>|z|)
(Intercept)	-7.6807848	0.1333161	-57.6133418	0.0000000
species2	-0.3410196	0.0543925	-6.2696075	0.0000000
species3	-0.4943257	0.0809380	-6.1074608	0.0000000
dbh	-0.0741110	0.0043993	-16.8462490	0.0000000
log(basalarea + 0.5)	1.0774641	0.0478825	22.5022435	0.0000000
harvennus_yli5vTRUE	0.0087776	0.0848957	0.1033928	0.9176512
snowload	0.0290434	0.0008957	32.4264434	0.0000000
relelev_1km	0.0283242	0.0023644	11.9796878	0.0000000
dem	0.0038793	0.0003447	11.2549892	0.0000000

Table 3: AIC values for models with and without variable "Thinning, > 5 years" (harvennus_yli5v)

	df	AIC
snowglm_with	9	20760.39
snowglm_without	8	20758.40

3. Precommercial thinning, > 5 years

Variable describes whether precommercial thinning has been carried out at the stand more than five years ago (TRUE/FALSE).

Table 4: Model with variable "Precommercial thinning, > 5 years" (ensiharvennus_yli5v)

	Estimate	Std. Error	z value	Pr(>|z|)
(Intercept)	-7.6835003	0.1333067	-57.6377688	0.0000000
species2	-0.3390768	0.0544250	-6.2301692	0.0000000
species3	-0.4929056	0.0809425	-6.0895756	0.0000000
dbh	-0.0743611	0.0043538	-17.0797438	0.0000000
log(basalarea + 0.5)	1.0781589	0.0478272	22.5427833	0.0000000
ensiharvennus_yli5vTRUE	0.0900984	0.0995052	0.9054644	0.3652194
snowload	0.0290292	0.0008957	32.4090946	0.0000000
relelev_1km	0.0282868	0.0023647	11.9622755	0.0000000

	Estimate	Std. Error	z value	Pr(>|z|)
dem	0.0038899	0.0003442	11.3026707	0.0000000

Table 5: AIC values for models with and without variable "Pre-commercial thinning, > 5 years" (ensiharvennus_yli5v)

	df	AIC
snowglm_with_yli5v	9	20759.6
snowglm_without	8	20758.4

4. Tending of seedling stands, > 5 years

Variable describes whether tending of seedling stand has been carried out at the stand more than five years ago (TRUE/FALSE).

Table 6: Model with variable "Tending of seedling stand, > 5 years" (taimihoito_yli5v)

	Estimate	Std. Error	z value	Pr(>|z|)
(Intercept)	-7.6613039	0.1328692	-57.660477	0.0000000
species2	-0.3375008	0.0543976	-6.204327	0.0000000
species3	-0.4910299	0.0809519	-6.065703	0.0000000
dbh	-0.0766650	0.0044058	-17.400812	0.0000000
log(basalarea + 0.5)	1.0913073	0.0478653	22.799540	0.0000000
taimihoito_yli5vTRUE	-0.3319052	0.1062829	-3.122846	0.0017911
snowload	0.0290211	0.0008961	32.386015	0.0000000
relelev_1km	0.0285544	0.0023641	12.078324	0.0000000
dem	0.0038746	0.0003434	11.281679	0.0000000

Table 7: AIC values for models with and without variable "Tending of seedling stand, > 5 years" (taimihoito_yli5v)

	df	AIC
snowglm_with2	9	20749.76
snowglm_without	8	20758.40

5. Average tree height

The variable describes the average tree height of the stand (dm).

Table 8: Model with variable "Tree height" (keskipit)

	Estimate	Std. Error	z value	Pr(>|z|)
(Intercept)	-7.6809409	0.1307901	-58.727217	0
species2	-0.3790882	0.0547601	-6.922711	0

	Estimate	Std. Error	z value	Pr(>|z|)
species3	-0.6434476	0.0835773	-7.698831	0
dbh	-0.1367818	0.0095640	-14.301765	0
log(basalarea + 0.5)	0.9325585	0.0500466	18.633819	0
keskipit	0.0095572	0.0012630	7.567300	0
snowload	0.0290278	0.0008921	32.539084	0
relelev_1km	0.0280500	0.0023792	11.789502	0
dem	0.0048769	0.0003767	12.946961	0

Table 9: AIC values for models with and without variable "Tree height" (keskipit)

	df	AIC
snowglm_with	9	20702.31
snowglm_without	8	20758.40

Table 10: Variation inflation factors (GVIF) for model with variable "Tree height" (keskipit)

	GVIF	Df	GVIF^(1/(2*Df))
species	1.119909	2	1.028716
dbh	8.290128	1	2.879258
log(basalarea + 0.5)	2.110775	1	1.452851
keskipit	9.902194	1	3.146775
snowload	1.180355	1	1.086442
relelev_1km	1.054602	1	1.026938
dem	1.476089	1	1.214944

Table 11: Model with variable "Tree height" (keskipit) while excluding DBH.

	Estimate	Std. Error	z value	Pr(>|z|)
(Intercept)	-7.5311012	0.1318312	-57.126832	0.00e+00
species2	-0.3465196	0.0545486	-6.352496	0.00e+00
species3	-0.3309589	0.0803161	-4.120706	3.78e-05
log(basalarea + 0.5)	0.9772503	0.0512775	19.058072	0.00e+00
keskipit	-0.0069564	0.0005808	-11.977487	0.00e+00
snowload	0.0292368	0.0008934	32.724019	0.00e+00
relelev_1km	0.0272636	0.0023391	11.655494	0.00e+00
dem	0.0027967	0.0003290	8.501453	0.00e+00

Table 12: Model with variable DBH while excluding "Tree height" (keskipit).

	Estimate	Std. Error	z value	Pr(>|z|)
(Intercept)	-7.6801166	0.1331415	-57.683872	0

	Estimate	Std. Error	z value	Pr(>|z|)
species2	-0.3411606	0.0543756	-6.274153	0
species3	-0.4944811	0.0809239	-6.110442	0
dbh	-0.0740317	0.0043307	-17.094747	0
log(basalarea + 0.5)	1.0771217	0.0477597	22.552942	0
snowload	0.0290449	0.0008956	32.431754	0
relelev_1km	0.0283292	0.0023638	11.984654	0
dem	0.0038766	0.0003437	11.280454	0

Table 13: AIC values for models with either Tree height (keskipit) or DBH included.

	df	AIC
snowglm_keskipit	8	20928.56
snowglm_dbh	8	20758.40

Table 14: Variation inflation factors (GVIF) for model with variable Tree height" (keskipit) while excluding DBH.

	GVIF	Df	GVIF^(1/(2*Df))
species	1.035841	2	1.008842
log(basalarea + 0.5)	2.018151	1	1.420616
keskipit	2.034260	1	1.426275
snowload	1.186678	1	1.089348
relelev_1km	1.062092	1	1.030578
dem	1.281239	1	1.131918

Table 15: Variation inflation factors (GVIF) for model with variable DBH while excluding "Tree height" (keskipit).

	GVIF	Df	GVIF^(1/(2*Df))
species	1.042045	2	1.010350
dbh	1.736970	1	1.317941
log(basalarea + 0.5)	1.774385	1	1.332061
snowload	1.182050	1	1.087221
relelev_1km	1.058808	1	1.028984
dem	1.295870	1	1.138363

6. Site type

Site type "FERTILE" or "POOR", reclassification of site type classes in the Finnish NFI (classes 1-3 = FERTILE, classes > 3 = POOR).

Table 16: Model with variable "Site type" (kptyyp_2class)

	Estimate	Std. Error	z value	Pr(>|z|)
(Intercept)	-7.6708986	0.1411311	-54.3530000	0.0000000
species2	-0.3453836	0.0584837	-5.9056382	0.0000000
species3	-0.4986814	0.0837127	-5.9570607	0.0000000
dbh	-0.0740416	0.0043304	-17.0980150	0.0000000
log(basalarea + 0.5)	1.0757593	0.0482355	22.3022110	0.0000000
kptyyp_2classPOOR	-0.0096050	0.0490838	-0.1956861	0.8448558
snowload	0.0290470	0.0008957	32.4299531	0.0000000
relelev_1km	0.0282998	0.0023685	11.9482003	0.0000000
dem	0.0038734	0.0003440	11.2604828	0.0000000

Table 17: AIC values for models with and without variable "Site type" (kptyyp_2class)

	df	AIC
snowglm_with	9	20760.36
snowglm_without	8	20758.40

7. Interactions: Species x DBH or Basal area

Interactions between two predictors:

- Species x DBH
- Species x Basal area

Table 18: Model with interaction Species x DBH

	Estimate	Std. Error	z value	Pr(>|z|)
(Intercept)	-7.6890780	0.1394996	-55.119006	0.0000000
species2	-0.5927490	0.1407292	-4.211982	0.0000253
species3	0.6395325	0.2183468	2.928975	0.0034008
dbh	-0.0746229	0.0050388	-14.809710	0.0000000
log(basalarea + 0.5)	1.0833878	0.0479459	22.596030	0.0000000
snowload	0.0291418	0.0008985	32.432293	0.0000000
relelev_1km	0.0281579	0.0023666	11.897829	0.0000000
dem	0.0038479	0.0003437	11.195885	0.0000000
species2:dbh	0.0139089	0.0073270	1.898313	0.0576548
species3:dbh	-0.0877992	0.0165816	-5.294979	0.0000001

Table 19: Model with interaction Species x Basal area

	Estimate	Std. Error	z value	Pr(>|z|)
(Intercept)	-7.9718471	0.1597659	-49.8970604	0.0000000
species2	-0.2182899	0.2551237	-0.8556238	0.3922059
species3	1.8375482	0.2872057	6.3980212	0.0000000

	Estimate	Std. Error	z value	Pr(>|z|)
dbh	-0.0761704	0.0043713	-17.4251090	0.0000000
log(basalarea + 0.5)	1.1856997	0.0562248	21.0885598	0.0000000
snowload	0.0291187	0.0008965	32.4804245	0.0000000
relelev_1km	0.0281458	0.0023636	11.9079099	0.0000000
dem	0.0039801	0.0003446	11.5489101	0.0000000
species2:log(basalarea + 0.5)	-0.0450562	0.0856941	-0.5257793	0.5990416
species3:log(basalarea + 0.5)	-0.8526833	0.1055349	-8.0796311	0.0000000

Table 20: AIC values for models with interaction Species x BA, with interaction Species x DBH and without interactions.

	df	AIC
snowglm_withBA	10	20710.78
snowglm_withDBH	10	20725.53
snowglm_without	8	20758.40

8. North boreal zone and interactions

Adding the north boreal zone ('north') into the model.

8.1 North

Table 21: Model with variable 'north' describing if plot located in north boreal zone.

	Estimate	Std. Error	z value	Pr(>|z|)
(Intercept)	-7.7207920	0.1329452	-58.075010	0.0000000
species2	-0.3473889	0.0544970	-6.374455	0.0000000
species3	-0.4952526	0.0809362	-6.119046	0.0000000
dbh	-0.0737315	0.0043357	-17.005868	0.0000000
log(basalarea + 0.5)	1.0816260	0.0482313	22.425796	0.0000000
snowload	0.0289589	0.0008986	32.227654	0.0000000
relelev_1km	0.0280042	0.0023811	11.761227	0.0000000
dem	0.0041480	0.0004152	9.990217	0.0000000
north	-0.0739752	0.0663517	-1.114895	0.2648954

Table 22: AIC values for models with and without variable 'north'.

	df	AIC
snowglm_without	8	20757.61
snowglm_with	9	20758.36

8.2 North x snowload

Testing interaction with snowload, as northern trees, adapted to heavy snowloads, could response to snowload differently.

Table 23: Model with interaction north x snowload

	Estimate	Std. Error	z value	Pr(>|z|)
(Intercept)	-7.7048499	0.1335721	-57.683082	0.0000000
species2	-0.3489004	0.0544964	-6.402259	0.0000000
species3	-0.4957242	0.0809140	-6.126555	0.0000000
dbh	-0.0738949	0.0043380	-17.034431	0.0000000
log(basalarea + 0.5)	1.0818972	0.0482135	22.439695	0.0000000
snowload	0.0286110	0.0009489	30.152055	0.0000000
relelev_1km	0.0279844	0.0023832	11.742412	0.0000000
dem	0.0041992	0.0004177	10.052605	0.0000000
north	-0.3232467	0.2235972	-1.445665	0.1482712
snowload:north	0.0034789	0.0029661	1.172865	0.2408501

Table 24: AIC values for models with and without interaction north x species

	df	AIC
snowglm_without	8	20757.61
snowglm_with	10	20758.99

8.3 North x DBH

Testing interaction with DBH, as northern trees, adapted to heavy snowloads, could have different pattern of DBH effect on damage probability.

Table 25: Model with interaction north x DBH

	Estimate	Std. Error	z value	Pr(>|z|)
(Intercept)	-7.6770374	0.1354296	-56.686537	0.0000000
species2	-0.3506124	0.0544991	-6.433363	0.0000000
species3	-0.4980986	0.0809638	-6.152114	0.0000000
dbh	-0.0763828	0.0046608	-16.388475	0.0000000
log(basalarea + 0.5)	1.0844146	0.0482347	22.482029	0.0000000
snowload	0.0289710	0.0008988	32.234397	0.0000000
relelev_1km	0.0279410	0.0023786	11.746599	0.0000000
dem	0.0040873	0.0004170	9.801631	0.0000000
north	-0.2778253	0.1465753	-1.895444	0.0580336
dbh:north	0.0131626	0.0083709	1.572416	0.1158540

Table 26: AIC values for models with and without interaction north x DBH

	df	AIC
snowglm_without	8	20757.61
snowglm_with	10	20757.88

8.4 North x Species

Testing interaction with DBH, as different adaptations to northern conditions are known between species, morphological adaptation being especially clear in Norway spruce.

Table 27: Model with interaction north x species

	Estimate	Std. Error	z value	Pr(>|z|)
(Intercept)	-7.7690642	0.1331775	-58.3361554	0.0000000
species2	-0.2531935	0.0578732	-4.3749697	0.0000121
species3	-0.4178832	0.0851977	-4.9048672	0.0000009
dbh	-0.0727090	0.0043321	-16.7838777	0.0000000
log(basalarea + 0.5)	1.0756412	0.0481114	22.3572976	0.0000000
snowload	0.0289123	0.0008991	32.1558917	0.0000000
relelev_1km	0.0273759	0.0023876	11.4657034	0.0000000
dem	0.0043324	0.0004181	10.3625481	0.0000000
north	0.0213918	0.0687567	0.3111231	0.7557071
species2:north	-0.7517200	0.1862165	-4.0368058	0.0000542
species3:north	-0.5686186	0.2803981	-2.0278977	0.0425707

Table 28: AIC values for models with and without interaction north x DBH

	df	AIC
snowglm_without	8	20757.61
snowglm_with	11	20740.17

9. Long term snowload x species

Testing the interaction between long term snowload (15 years average of winter max) and species, as Norway spruce shows clearer morphological differentiation with latitude and could therefore show species-specific reponse to long-term snowload.

Table 29: Model with interaction Long term snowload x species

	Estimate	Std. Error	z value	Pr(>|z|)
(Intercept)	-7.2849719	0.1841717	-39.5553273	0.0000000
species_spruce	0.1068927	0.2860188	0.3737262	0.7086080
species_other	0.7234707	0.4648012	1.5565164	0.1195854
dbh	-0.0732843	0.0043957	-16.6719259	0.0000000
log(basalarea + 0.5)	1.1190323	0.0484103	23.1155616	0.0000000

	Estimate	Std. Error	z value	Pr(>|z|)
snowload_longterm	-0.0243462	0.0048049	-5.0669548	0.0000004
snowload	0.0325340	0.0010532	30.8897068	0.0000000
relelev_1km	0.0270099	0.0023912	11.2953546	0.0000000
dem	0.0050485	0.0003924	12.8652716	0.0000000
species_other:dbh	-0.0920792	0.0163444	-5.6336874	0.0000000
species_spruce:snowload_longterm	-0.0130581	0.0076964	-1.6966466	0.0897635
species_other:snowload_longterm	-0.0005436	0.0111923	-0.0485689	0.9612629

Table 30: AIC values for models with and without interaction Long term snowload x species

	df	AIC
snowglm_without	10	20692.53
snowglm_with	12	20693.62

10. Species composition

The effect of species composition was studied with three different variables:

- number of species
- BA proportion of most abundant species (most abundant here meaning the species with largest basal area)
- Shannon diversity index (calculated from % of basal area)

Table 31: Model with number of tree species (n_species).

	Estimate	Std. Error	z value	Pr(>|z|)
(Intercept)	-7.0574239	0.1313806	-53.7173884	0.0000000
species_spruce	-0.2681443	0.0586793	-4.5696554	0.0000049
species_other	0.7433953	0.2149934	3.4577594	0.0005447
dbh	-0.0728983	0.0043807	-16.6406130	0.0000000
log(basalarea + 0.5)	1.1190169	0.0489719	22.8501796	0.0000000
log(n_species)	-0.1060911	0.0467952	-2.2671358	0.0233819
snowload_anomaly	0.0330034	0.0009908	33.3092338	0.0000000
relelev_1km	0.0256751	0.0024017	10.6904942	0.0000000
dem	0.0059132	0.0004028	14.6797453	0.0000000
north	-0.0522483	0.0690748	-0.7564027	0.4494078
species_other:dbh	-0.0922254	0.0163765	-5.6315877	0.0000000
species_spruce:north	-0.7439038	0.1860639	-3.9981099	0.0000639

Table 32: Model with proportion of BA covered by the most abundant species (max_sp_percent).

	Estimate	Std. Error	z value	Pr(>|z|)
(Intercept)	-7.3518540	0.1749239	-42.0288627	0.0000000

	Estimate	Std. Error	z value	Pr(>|z|)
species_spruce	-0.2660797	0.0587368	-4.5300340	0.0000059
species_other	0.7380216	0.2143258	3.4434572	0.0005743
dbh	-0.0728918	0.0043809	-16.6385639	0.0000000
log(basalarea + 0.5)	1.1140774	0.0488026	22.8282622	0.0000000
max_sp_percent	0.2925138	0.1239580	2.3597807	0.0182857
snowload_anomaly	0.0329717	0.0009897	33.3145282	0.0000000
relelev_1km	0.0255505	0.0024013	10.6401676	0.0000000
dem	0.0059055	0.0004028	14.6607314	0.0000000
north	-0.0477269	0.0689040	-0.6926569	0.4885249
species_other:dbh	-0.0909531	0.0163410	-5.5659568	0.0000000
species_spruce:north	-0.7336440	0.1862241	-3.9395766	0.0000816

Table 33: Model with Shannon diversity index (shannon_div).

	Estimate	Std. Error	z value	Pr(>|z|)
(Intercept)	-7.1817792	0.1360826	-52.7751643	0.0000000
species_spruce	-0.2643695	0.0585866	-4.5124552	0.0000064
species_other	0.7476963	0.2146353	3.4835655	0.0004948
dbh	-0.0727750	0.0043761	-16.6300508	0.0000000
log(basalarea + 0.5)	1.1190954	0.0488620	22.9031634	0.0000000
log(shannon_div + 1e-04)	-0.0157360	0.0054827	-2.8701187	0.0041032
snowload_anomaly	0.0330205	0.0009905	33.3364265	0.0000000
relelev_1km	0.0255899	0.0024028	10.6502298	0.0000000
dem	0.0059209	0.0004030	14.6917770	0.0000000
north	-0.0536816	0.0690004	-0.7779896	0.4365751
species_other:dbh	-0.0922571	0.0163489	-5.6430204	0.0000000
species_spruce:north	-0.7316969	0.1861833	-3.9299818	0.0000850

Table 34: AIC values of models with species community variables.

	df	AIC
snowglm_without	11	20674.88
snowglm_n_species	12	20671.75
snowglm_max_sp_percent	12	20671.25
snowglm_shannon	12	20668.74
